# Stimulation of fat oxidation in rat muscle by unacylated ghrelin persists for 2–3 hours, but is independent of fatty acid transporter translocation

**DOI:** 10.14814/phy2.15815

**Published:** 2023-09-19

**Authors:** Evan M. Hoecht, Joshua M. Budd, Nicole M. Notaro, Graham P. Holloway, David J. Dyck

**Affiliations:** ^1^ Department of Human Health and Nutritional Sciences University of Guelph Guelph Ontario Canada

**Keywords:** FABPpm, FAT/CD36, fatty acid oxidation, skeletal muscle, unacylated ghrelin

## Abstract

While a definitive mechanism‐of‐action remains to be identified, recent findings indicate that ghrelin, particularly the unacylated form (UnAG), stimulates skeletal muscle fatty acid oxidation. The biological importance of UnAG‐mediated increases in fat oxidation remains unclear, as UnAG peaks in the circulation before mealtimes, and decreases rapidly during the postprandial situation before increases in postabsorptive circulating lipids. Therefore, we aimed to determine if the UnAG‐mediated stimulation of fat oxidation would persist long enough to affect the oxidation of meal‐derived fatty acids, and if UnAG stimulated the translocation of fatty acid transporters to the sarcolemma as a mechanism‐of‐action. In isolated soleus muscle strips from male rats, short‐term pre‐treatment with UnAG elicited a persisting stimulus on fatty acid oxidation 2 h after the removal of UnAG. UnAG also caused an immediate phosphorylation of AMPK, but not an increase in plasma membrane FAT/CD36 or FABPpm. There was also no increase in AMPK signaling or increased FAT/CD36 or FABPpm content at the plasma membrane at 2 h which might explain the sustained increase in fatty acid oxidation. These findings confirm UnAG as a stimulator of fatty acid oxidation and provide evidence that UnAG may influence the handling of postprandial lipids. The underlying mechanisms are not known.

## 1 INTRODUCTION

Ghrelin is classically known as an orexigenic hormone. However, a growing body of research has identified a role for ghrelin in the regulation of peripheral tissue glucose and fatty acid metabolism. Ghrelin exists in two isoforms, acylated (AG) and unacylated (UnAG) ghrelin. AG is responsible for the hormone's orexigenic activity (Kojima et al., 1999; Nakazato et al., 2001; Wren et al., 2000). UnAG has a higher concentration in the circulation (Mizutani et al., 2009; Tong et al., 2013) and has been shown to regulate metabolism in peripheral tissues such as skeletal muscle (Barazzoni et al., 2005, 2007, 2014, 2017; Cervone et al., 2019, 2020; Kraft et al., 2019; Lovell et al., 2022). Both isoforms peak in the circulation immediately prior to entrained mealtimes, followed by a rapid reduction to near baseline (i.e., within 30–60 min) upon the consumption of a meal (Cummings et al., 2001; Liu et al., 2008; Tschop et al., 2001).

The overexpression of UnAG has been shown to preserve insulin signaling and reduce inflammation in the gastrocnemius muscle of mice fed a high‐fat diet for 16 weeks (Gortan Cappellari et al., 2016). It is possible that this protective effect is due in part to the ability of UnAG to stimulate fatty acid oxidation. Our lab has previously shown that UnAG can acutely increase fatty acid oxidation in isolated rat soleus muscle (Cervone et al., 2020; Kraft et al., 2019; Lovell et al., 2022). Importantly, this increase in fatty acid oxidation prevents the impairment of insulin‐stimulated glucose uptake normally observed in high‐palmitate conditions (Cervone et al., 2020; Lovell et al., 2022). This is consistent with earlier findings that triglyceride accumulation and impaired glucose uptake in primary cultured rat myoblasts incubated with high‐fat were minimized by co‐incubating with AG (Han et al., 2015). Activation of the AMPK/ACC axis is generally considered as a signaling mechanism to increase fatty acid entry into the mitochondrion and its subsequent oxidation. However, results in muscle have been inconsistent and only a few studies have examined UnAG specifically (Cervone & Dyck, 2017; Kraft et al., 2019). AG has been shown to stimulate AMPK in myoblasts (Han et al., 2015), muscle (Kraft et al., 2019) and heart (Kola et al., 2005, 2013).

Skeletal muscle represents ~40% of total body mass and is a significant tissue in the clearance of postprandial substrates. Insulin is the predominant hormonal signal for the uptake of glucose. While the absorption of glucose into the circulation occurs within minutes of consumption, the entry of lipids into the circulation occurs ~2 h after consumption (Dubois et al., 1998; Panzoldo et al., 2011; Snook et al., 2016). Insulin is a known stimulus for the acute translocation of the fatty acid transporter FAT/CD36 to the cell surface (Luiken et al., 2002; Snook et al., 2016; Stefanyk et al., 2012; van Oort et al., 2008). However, this translocation is transient and no longer present after 1 h (Snook et al., 2016; van Oort et al., 2008). This suggests that insulin is not the primary signal mediating postprandial fatty acid uptake into muscle.

With recent findings that UnAG can stimulate fatty acid oxidation in skeletal muscle, it is relevant to question whether this stimulation persists long enough, that is, 2–3 h to affect the oxidation of meal‐derived fatty acids. Furthermore, it is unknown whether part of the mechanism underlying the ability of UnAG to stimulate fatty acid oxidation involves the translocation of fatty acid transporters to the sarcolemma. To our knowledge, no study has examined the ability of ghrelin to induce the translocation of fatty acid transporters. Therefore, the objective of this study was to determine if UnAG induces a persistent stimulation of fatty acid oxidation (i.e., for 2–3 h), and whether fatty acid transporter translocation to the sarcolemma is involved. Finally, we assessed the ability of UnAG to influence in vivo whole‐body substrate metabolism for up to 3 h post‐injection.

## 2 METHODS

### 2.1 Animals

All procedures were approved by the Animal Care Committee at the University of Guelph and followed Canadian Council of Animal Care guidelines. For all experiments, male Sprague–Dawley rats were obtained from Charles River Laboratories Canada at approximately 6‐weeks of age (151–175 g). Rats were given access ad libitum to chow diet (Tekland 2018 laboratory diet, Envigo) and water. Rats were acclimated for a minimum of 1 week prior to experiments and housed in groups of 3–4 at 22–24°C with a 12 h reverse light:dark cycle. To avoid confounding high levels of circulating ghrelin, prior to all surgical procedures or interventions, rats were fasted overnight with a brief re‐introduction of food (30 min) at the beginning of their dark cycle and approximately 2–3 h prior to tissue removal or intervention. We have previously confirmed that this prevents high levels of endogenous circulating ghrelin that typically occurs with fasting (Cervone et al., 2019). Prior to tissue removal, rats were anesthetized with a intraperitoneal bolus of pentobarbital sodium (6 mg/100 g body mass).

### 2.2 Materials and reagents

Reagents, molecular weight markers, and nitrocellulose membranes for all western blotting experiments were purchased from BioRad Laboratories. All BCA assay reagents were purchased from Fisher Scientific. The following antibodies were provided as gifts: FAT/CD36 (M025) from Dr. N.N. Tandon (Otsuka Maryland Medical Laboratories), FABPpm from J. Calles‐Escandon (Wake Forest University), and MCT‐1 from Dr. H Hatta (Tokyo University). Insulin (Humulin rDNA origin) and FreeStyle Lite blood glucose monitoring system (Abbott Diabetes Care Canada) were purchased from Shoppers Drug Mart. All other reagents are listed in Table 1.

**TABLE 1:** Listing of reagents used in experiments and analyses.

Reagent	Source	Catalogue number
Alpha‐tubulin	Cell Signaling Technology (Danvers, MA, USA)	2144S
ACC antibody	Cell Signaling Technology (Danvers, MA, USA)	3676S
Phospho‐ACC Ser79 antibody	Cell Signaling Technology (Danvers, MA, USA)	11818S
5‐amino‐1‐β‐D‐ribofuranosyl‐1H‐imidazole‐4‐carboxamide (AICAR)	Cayman Chemical (Ann Arbor, MI, USA)	10,010,241
Ammonium persulfate	Millipore Sigma (Oakville, ON, Canada)	A3678
AMPK antibody	Cell Signaling Technology (Danvers, MA, USA)	2532S
Phospho‐AMPK Thr172 antibody	Cell Signaling Technology (Danvers, MA, USA)	2535S
Aprotinin	BioShop, Burlington, ON, Canada	APR600
Benzethonium hydroxide solution	Millipore Sigma (Oakville, ON, Canada)	B2156
Bovine serum albumin (BSA)	Millipore Sigma (Oakville, ON, Canada)	A7030
Collagenase, Type IV	Millipore Sigma (Oakville, ON, Canada)	C0773
Dimethyl sulfoxide	Millipore Sigma (Oakville, ON, Canada)	D8418
Disodium dihydrate ethylenediamine tetraacetic acid (EDTA)	BioShop, Burlington, ON, Canada	EDT001
Dulbecco's modified Eagle's media (DMEM)	Millipore Sigma (Oakville, ON, Canada)	D5030
D‐glucose	Millipore Sigma (Oakville, ON, Canada)	G‐8270
Total plasma ghrelin ELISA kit	Millipore Sigma (Oakville, ON, Canada)	EZRGRT‐91K
Glucose transporter 4 (GLUT4)	Cell Signaling Technology (Danvers, MA, USA)	2213S
Goat anti‐mouse	BioRad Laboratories (Mississauga, ON, Canada)	1,706,516
Goat anti‐rabbit	BioRad Laboratories (Mississauga, ON, Canada)	170–6515
Histodenz	Millipore Sigma (Oakville, ON, Canada)	D2158
Invitrogen NP40 cell lysis buffer	Life Technologies (Burlington, ON, Canada)	FNN0021
3‐[N‐morpholino]propanesulfonic acid (MOPS)	BioShop, Burlington, ON, Canada	MOPP001
Palmitic acid	Millipore Sigma (Oakville, ON, Canada)	P0500
^14C^palmitic acid	American Radiolabeled Chemicals Inc. (St. Louis, MO, USA)	0172A
Percoll	Amersham Biosciences (Amersham, United Kingdom)	17–0891‐01
Phenylmethylsulfonyl fluoride (PMSF)	Millipore Sigma (Oakville, ON, Canada)	P7626
Protease inhibitor cocktail	Millipore Sigma (Oakville, ON, Canada)	P8340
Potassium chloride	BioShop, Burlington, ON, Canada	POC001
SERCA2 antibody	Abcam (Cambridge, UK)	Ab2861
Sodium bicarbonate	Millipore Sigma (Oakville, ON, Canada)	S5761
Sodium chloride solution, sterile	Millipore Sigma (Oakville, ON, Canada)	S8776
Unacylated rat ghrelin, synthetic	Bachem (Torrence, CA, USA)	H‐6264
Western lighting plus enhanced chemiluminescence (ECL)	PerkinElmer Canada (Woodbridge, ON, Canada)	NEL105001EA

### 2.3 Treatments

Fatty acid oxidation in response to UnAG (150 ng/mL) for up to 3 h was conducted in vitro using isolated soleus muscle strips obtained from one set of animals. Signaling measurements were conducted in isolated soleus strips from a second set of animals. To determine the effect of UnAG on sarcolemmal fatty acid transporter content, rats were injected with either UnAG (1 mg/kg body weight) or saline (control). Insulin (1.5 U/kg body weight) was injected as a positive control. Red gastrocnemius and quadriceps muscles were sampled at 30 min and 2 h post‐injection. Different animals were used for each timepoint. Rats were injected with pentobarbital sodium ~20–25 min prior to tissue removal. Soleus muscle from rats were also excised, rapidly frozen in liquid nitrogen, and stored at −70°C for later analysis as a whole muscle control to check the quality of GSV preps. Whole body RER in response to UnAG (1 mg/kg) and insulin (1.5 U/kg) injections were determined in vivo using a Comprehensive Lab Monitoring System (CLAMS). Details of these treatments are presented below.

#### 2.3.1 Isolated soleus incubations for assessment of palmitate oxidation

Assessment of palmitate oxidation was as previously reported from our laboratory using ^14^C‐palmitate (Cervone et al., 2020; Kraft et al., 2019; Lovell et al., 2022). Briefly, soleus muscle strips were excised tendon‐to‐tendon and placed in pre‐gassed vials (95% O_2_; 5% CO_2_) in a warmed (30°C) shaking water bath. Each soleus muscle was gently separated into four sections ~20–25 mg, allowing for 4 separate experimental treatments. Vials contained DMEM supplemented with sodium bicarbonate, 8 mM glucose, 4% bovine albumin, 1 mM palmitate and 50μU/mL insulin. Muscles were incubated in this media for 30–45 min to allow for recovery after surgical removal. The same base media was used for the subsequent experimental conditions. All experimental incubations were for 3 h, and 14C‐palmitate oxidation was measured during the final (third) hour. Muscles were incubated in sealed Erlenmeyer flasks. The conditions were (i) control (no UnAG) for the entire 3 h; (ii) prolonged exposure to UnAG (150 ng/mL) during the second and third hours; (iii) late exposure to UnAG during the third hour only; and (iv) early exposure to UnAG during the first hour only. The experimental condition of primary interest was the final one in which we determined whether the stimulation of palmitate oxidation by UnAG can persist for up to 2 h after UnAG has been removed. The 2 h exposure to UnAG has previously been shown to increase palmitate oxidation (Cervone et al., 2020) and provided a positive control. The condition exposing muscle to UnAG in the final (third) hour was to ensure that the muscle could still be stimulated by UnAG after 3 h of incubation.

At the end of the incubation period, 250 μL benzethonium hydroxide was injected into an Eppendorf tube suspended from a rubber stopper to capture the ^14^CO_2_ released by the injection of 1 M sulfuric acid directly into the incubation media which still contained the muscle. The acidified flask was left for 2 h at room temperature. Following this, soleus strips were removed, tendons were trimmed, and muscle was reweighed. The tubes containing benzethonium hydroxide and trapped ^14^CO_2_ were placed in liquid scintillation vials with the addition of 5 mL of scintillation cocktail. Samples were quenched overnight in full darkness prior to quantification using a PerkinElmer Tri‐Carb LSC 4910 TR liquid scintillation counter. Samples were counted for 5 min each.

#### 2.3.2 Signaling

Intracellular skeletal muscle signaling was conducted using a separate set of animals, and incubation conditions similar to those for palmitate oxidation with the omission of the ^14C^‐palmitate. Briefly, soleus muscle strips were procured and placed in a recovery buffer (modified DMEM as previously indicated) for 30–45 min. After this initial period, muscle strips underwent one of the following experimental conditions: control (no UnAG, 15, 45, or 120 min), 2 mM AICAR (15, 45 min) or 150 ng/mL UnAG (15, 45, or 120 min). Muscle strips derived from one leg were incubated for the same duration (15, 45, or 120 min) and randomly assigned to one of either the control, AICAR or UnAG conditions. AMPK phosphorylation was analyzed at 15 min, and ACC phosphorylation at 45 min and 2 h. Fifteen‐minute and 45‐min timepoints were chosen to capture near‐maximal phosphorylation states of AMPK and ACC, respectively (Tomas et al., 2002). For the assessment of ACC phosphorylation with UnAG at 2 h, strips initially exposed to UnAG for 1 h and then transferred to a final vial without UnAG for another 2 h. Following incubations, soleus muscle strips were immediately blotted dry, rapidly frozen in liquid nitrogen, and stored at −70°C for western blot analysis.

#### 2.3.3 Sarcolemmal fatty acid transporter assessment

Sarcolemmal content of fatty acid transporters (FAT/CD36, FABPpm) in response to UnAG injections was assessed using giant sarcolemmal vesicles (GSV). Briefly, approximately 1 g of muscle was minced in ice‐cold KCl/MOPS with protease inhibitors PMSF and aprotinin, and Type IV collagenase to facilitate budding of the sarcolemma. Samples were incubated at 34°C for 90 min in a shaking water bath. Muscle tissue and medium were then filtered through a four‐layer cheesecloth with a 10 mM EDTA wash into graduated cylinders containing Percoll. Muscle was discarded and the resulting solution was mixed and pipetted below a HistoDenz layer in a 15 mL centrifuge tube to create a density gradient. A final layer of vesicle preparation medium (VPM; KCl/MOPS supplemented with PMSF) was added to the top of the HistoDenz layer to an end volume of 15 mL to complete a three‐layer density gradient. Samples were centrifuged at 60*g* for 45 min. Vesicles appeared at the Histodenz/VPM interface and were collected into Eppendorf tubes. Finally, samples were spun at 12000*g* for 5 min leaving a pellet containing GSVs. GSVs were resuspended in VPM and frozen at −70°C for future analysis.

#### 2.3.4 Western blotting

##### Skeletal muscle

Frozen soleus muscle (~20–30 mg) was placed into homogenization tubes with lysis beads (two per tube). Samples were immediately immersed in ice‐cold cell lysis buffer supplemented with PMSF and protease inhibitor cocktail. Muscle tissue was homogenized in Qiagen TissueLyser LT (cat. No. 85600), centrifuged at 1500*g* for 15 min at 4°C, and the supernatant was collected into a new tube. Protein concentration of the supernatant was determined by a bicinchoninic acid (BCA) assay to ensure equal loading of protein during gel electrophoresis. Protein (10 μg) was loaded into wells on a 10% acrylamide gels (7.5% for ACC) and separated based on molecular weights. Protein was then transferred for 1 h (1.5 h for ACC) at 100 V onto nitrocellulose membranes at 4°C and then blocked in 5% w/v non‐fat skim milk 1X TBST for 1 h at room temperature on a rocker. Following blocking, membranes were washed in 1X TBST and incubated overnight at 4°C in primary antibody (1:1000 dilution in 5% BSA or 5% non‐fat skim milk 1X TBST as per manufacturer directions) on a rocker. The following morning, membranes were washed in 1X TBST for 30 min (2 × 15 min) and incubated in secondary anti‐rabbit or anti‐mouse antibody depending on host species of primary (1:2000 dilution in 1% non‐fat skim milk 1X TBST) for 1 h. Membranes were washed again for 40 min (2 × 15 min in 1X TBST followed by 10 min in 1X TBS). Protein bands on membranes were visualized via ECL reagents and quantified using Alpha Innotech Software. Primary antibodies used for quantification of proteins included: phospho‐AMPK Thr127, total‐AMPK, phospho‐ACC Ser79, total‐ACC, and alpha‐tubulin as a loading control.

##### Giant sarcolemmal vesicles

BCA assay of GSVs suspended in KCl/MOPS was conducted to determine protein concentrations to allow for equal loading of protein during gel electrophoresis. GSV protein (10 μg) was loaded into wells on 10% acrylamide gels and protein was separated based on molecular weight. Protein was then transferred, blocked, washed, incubated in primary and secondary antibodies, and visualized identically to that of skeletal muscle samples described above. Primary antibodies used for quantification of GSV proteins included: FAT/CD36, FABPpm, GLUT4, SERCA2a, and MCT‐1 as a loading control.

#### 2.3.5 Comprehensive lab animal monitoring system (CLAMS)

A Comprehensive Lab Animal Monitoring System (CLAMS) was used for in vivo analysis of substrate utilization in rats. Rats were housed individually in CLAMS caging and acclimated for 15 h prior to any interventions. CLAMS temperature and lighting mirrored the housing conditions of our animal room. Experimental interventions were an intraperitoneal injection of either saline, insulin (1.5 U/kg) or UnAG (1 mg/kg). Immediately following injection, rats were returned to their assigned metabolic caging unit and respiratory data were collected for a 3‐h period (VCO_2_, VO_2_, RER). Three hours was chosen to allow analysis of the immediate period post‐injection and an extended period similar to our in vitro assessment of palmitate oxidation.

#### 2.3.6 Blood collection

Following hind‐limb skeletal muscle collection for GSV generation, blood was sampled directly from the exposed femoral artery. Blood glucose was determined using a Freestyle Lite glucometer. To measure circulating ghrelin concentrations, blood was collected in Eppendorf tubes containing EDTA (1 mg/mL) and aprotinin (~600–700 kIU/mL), placed on ice for 30 min, and then centrifuged for 5 min at 9500*g*. Supernatant was collected, acidified by 1 M HCl (100 μL/1 mL blood), and frozen at −70°C for future analysis. Plasma total ghrelin was measured using an ELISA kit (Millipore Sigma, Cat. No. EZRGRT‐91K).

### 2.4 Statistics

All statistical analysis was conducted in GraphPad Prism 9 software. For analysis of palmitate oxidation and all whole muscle western blot proteins (excluding 2‐h phospho‐ACC analysis), a repeated measures one‐way ANOVA was conducted. Statistical significance was set at *p* < 0.05. When statistical significance was found with the ANOVA, a Tukey's post hoc test was used to determine specific differences between groups. Two‐hour phospho‐ACC western blot, blood glucose, and serum UnAG were analyzed in their respective groupings using a paired *t*‐test. For analysis of GSV proteins a one‐way ANOVA was conducted. Statistical significance was tested using a Tukey's post hoc test. For analysis of treatment effects during CLAMS experiments, a two‐way ANOVA (treatment vs. time) was conducted. If statistical significance was found a Bonferroni post hoc test was used. In all cases an outlier test was conducted to identify and remove outliers from the datasets. Using the ROUT (robust regression and outlier) method in GraphPad Prism.

## 3 RESULTS

### 3.1 Stimulation of fatty acid oxidation by unacylated ghrelin persists for up to 3 h, ex vivo

**FIGURE 1:**
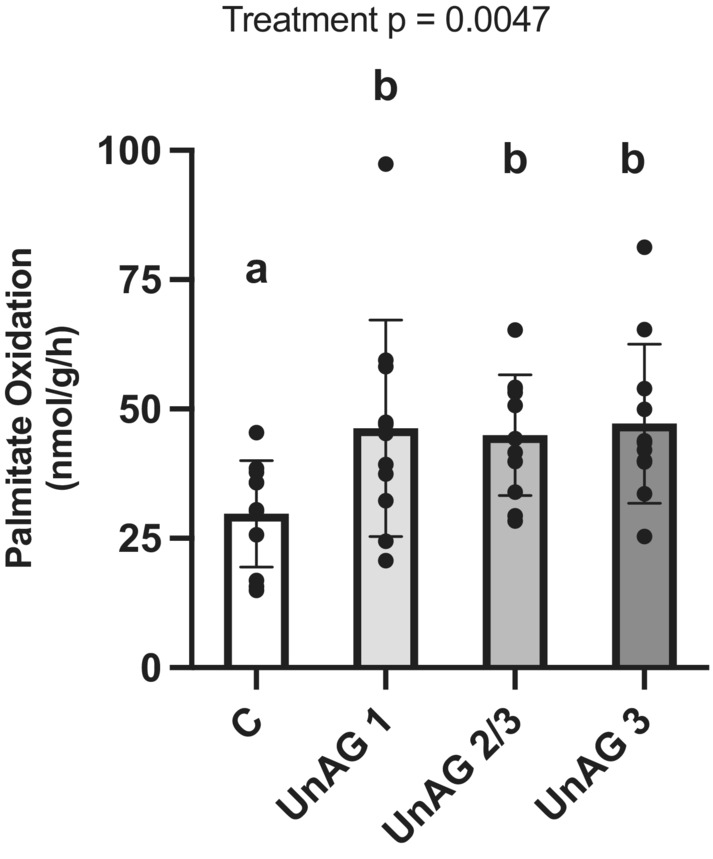
Soleus muscle palmitate oxidation in the presence of UnAG and up to 2 h after removal of UnAG from incubation media. C: control; UnAG 1: UnAG exposure during Hour 1 only; UnAG 2/3: UnAG exposure during Hours 2 and 3; UnAG 3: UnAG exposure during Hour 3 only. UnAG stimulated palmitate oxidation at all timepoints assessed. Data are expressed as mean ± SD (*n* = 11 per group). Data were analyzed using a repeated measures one‐way ANOVA and followed by a Tukey's post hoc test. Statistical significance was accepted at *p* < 0.05. Data sharing a letter are not statistically different.

UnAG stimulated palmitate oxidation (Figure 1) when present for both the final 1 h (*p* = 0.0096) and 2 h (*p* = 0.0032) of incubation. More specifically, to address one of the major aims of this study, an initial exposure to UnAG for 1 h resulted in a significant increase in palmitate oxidation (*p* = 0.0186) in the final (third) hour of incubation, that is, stimulation of fatty acid oxidation was sustained.

### 3.2 Immediate, but not persistent effects of unacylated ghrelin on fatty acid oxidation are related to stimulation of the AMPK/ACC axis

**FIGURE 2:**
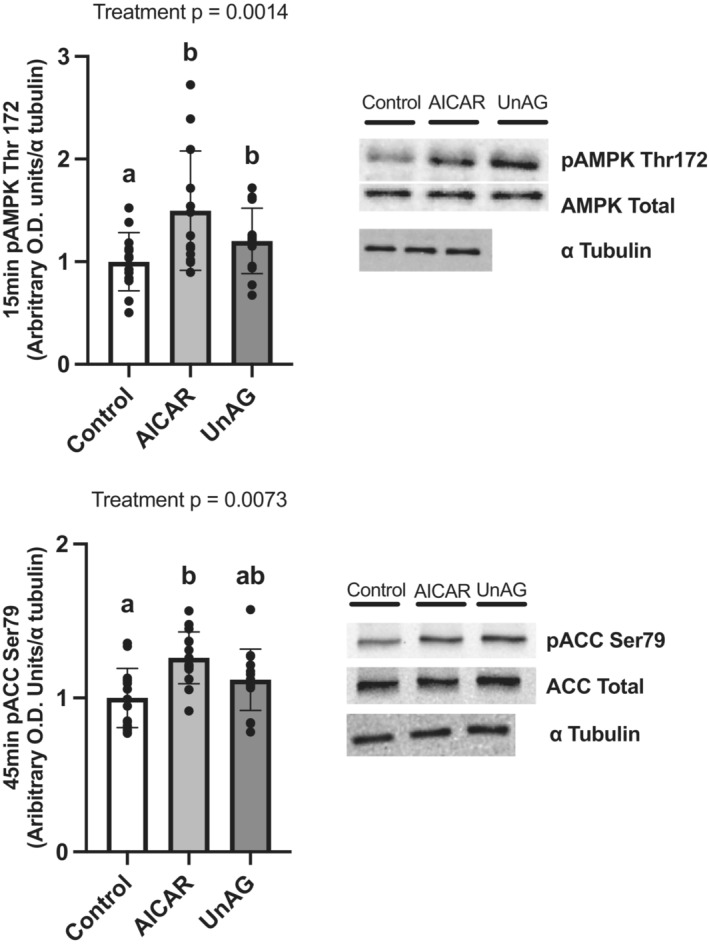
Phosphorylation of the AMPK/ACC axis at 15 and 45 min. The phosphorylation of AMPK at Thr172 (a) and ACC at Ser79 (b) after treatment with AICAR (2 mM) or UnAG (150 ng/mL). Data are expressed as mean ± SD. (AMPK, *n* = 13; ACC, *n* = 15). Data were analyzed using a repeated measures one‐way ANOVA and differences between groups were tested using a Tukey's post hoc test. Statistical significance was accepted at *p* < 0.05. Data sharing a letter are not statistically different.

**FIGURE 3:**
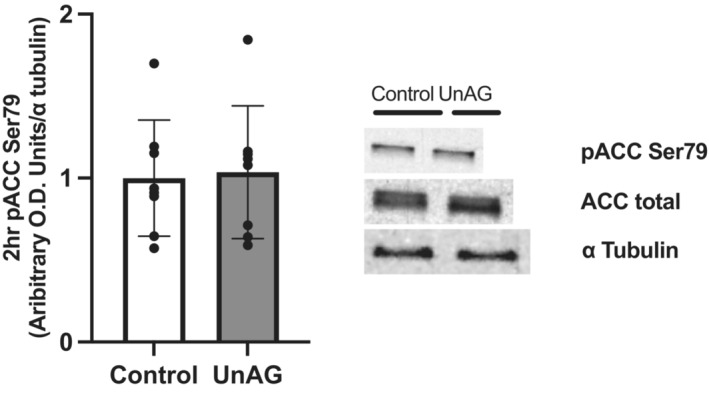
Ser79 phosphorylation of ACC 2 h after removal from incubation media. Data are expressed as mean ± SD (*n* = 8). The phosphorylation of ACC at Ser79 2 h after removal of UnAG from incubation media was not different from control with no treatment. Data were analyzed using a paired *t*‐test. Statistical significance was accepted at *p* < 0.05. No statistical differences were found.

Our lab has previously shown that UnAG can increase the phosphorylation of key stimulatory and inhibitory residues on AMPK and ACC, respectively (Barazzoni et al., 2007, 2017). AICAR served as a positive control and increased Thr172 phosphorylation of AMPK (Figure 2a) after 15 min (*p* = 0.0023) and Ser79 phosphorylation of ACC (Figure 2b) after 45 min of exposure (*p* < 0.0001). UnAG also increased Thr172 phosphorylation of AMPK after 15 min (*p* = 0.0343), but not Ser79 phosphorylation of ACC after 45 min (*p* = 0.225). We also tested the ability of UnAG to sustain Ser79 phosphorylation of ACC up to 2 h after the removal of UnAG from incubation media (Figure 3), which it did not (*p* = 0.835).

### 3.3 In vivo injection of insulin but not unacylated ghrelin induces translocation of GLUT4 to the sarcolemma of rat hind limb muscles at 30 min

Blood glucose was significantly decreased 30 min after insulin injection, and recovered to control levels following 2 h (Table 2). Blood glucose was unaffected by UnAG injection. We also confirmed an increase in circulating total ghrelin concentration following injection of UnAG. Plasma ghrelin concentration in saline‐injected rats ranged from 0.8 to 1.9 ng/mL. Thirty minutes after UnAG injection, concentrations were generally above the level of detection (>10 ng/mL).

**TABLE 2:** Blood glucose concentrations 30 min and 120 min following intraperitoneal injection of saline, insulin, or UnAG.

	Treatment		
	Saline (control)	Insulin (1.5 U/kg)	UnAG (1 mg/kg)
30 min post‐injection Blood glucose (mmol/L)	6.7 ± 1.2	2.6 ± 0.6*	6.5 ± 0.9
2 h post‐injection Blood glucose (mmol/L)	6.7 ± 1.1	5.8 ± 2.2	6.7 ± 1.2

*Note*: Data are reported as mean ± standard deviation of each group (*n* = 7–9). Data were analyzed by one‐way ANOVA for a given timepoint followed by a Tukey post hoc test as appropriate.

* *p* < 0.0001.

**FIGURE 4:**
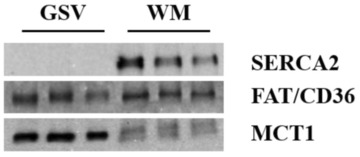
Quality of giant sarcolemmal vesicle preparation. *n* = 6–8. GSVs prepped were devoid of intracellular protein SERCA2. GSVs only contained protein concentrated on the sarcolemma (FAT/CD36 and MCT1). Whole muscle homogenate (WM) contained intracellular protein SERCA2, as well as FAT/CD36 and MCT1 to a lesser degree.

**FIGURE 5:**
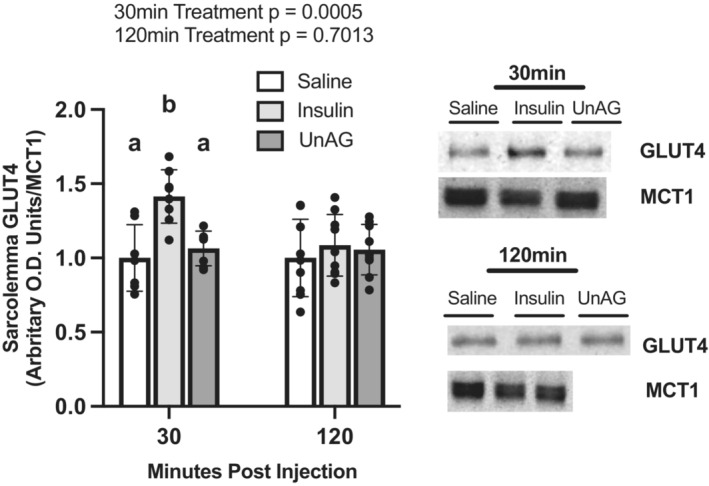
Isolated sarcolemma GLUT4 concentrations in response to saline, insulin, and UnAG injections. Data are expressed as mean ± SD (*n* = 7–9). Data were analyzed using an ordinary one‐way ANOVA at each timepoint; statistical significance was confirmed using a Tukey's post hoc test for a given timepoint. Statistical significance was accepted at *p* < 0.05. Data sharing a letter are not statistically different.

To confirm GSV preparation quality and that our model gave predicted results, GLUT4 translocation to the sarcolemma was determined at 30‐ and 120‐min post‐injection of insulin. These timepoints have previously shown a relative increase (30 min) and return to baseline (120 min) of sarcolemmal content of GLUT4 (39). We also sought to determine the quality of GSV preparation by checking for contamination with the intracellular protein SERCA2. Our results showed that SERCA2 was only detected in the whole muscle homogenate, but not GSVs (Figure 4). As expected, MCT1 was present in the whole muscle homogenate, but enriched in GSVs. Thirty minutes post‐insulin injection the concentration of GLUT4 at the sarcolemma (Figure 5) was increased relative to saline‐injected controls (*p* = 0.0052). At 120 min post‐injection, GLUT4 concentrations did not differ from saline‐injected controls (*p* = 0.6192). UnAG did not affect GLUT4 concentrations at the sarcolemma at either 30 or 120 min after injection relative to saline (30 min *p* = 0.7187; 120 min *p* = 0.8090).

### 3.4 Unacylated ghrelin does not induce translocation of FAT/CD36 or FABPpm to the sarcolemma

#### 3.4.1 Fat/CD36

**FIGURE 6:**
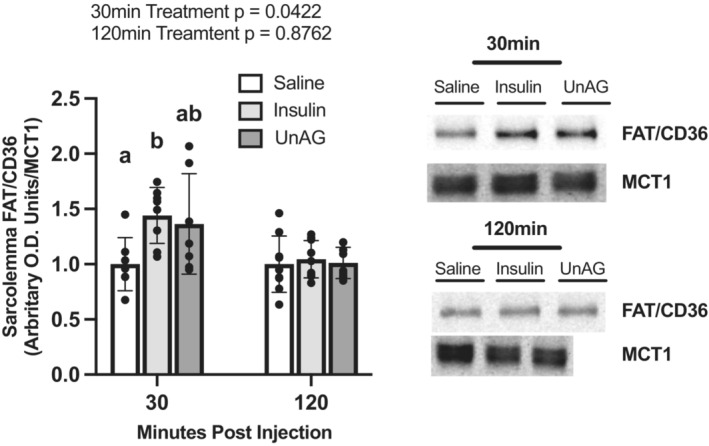
Isolated sarcolemma FAT/CD36 concentrations in response to saline, insulin, and UnAG injections at 30 min and 120 min post‐injection. Data are expressed as mean ± SD (*n* = 7–9). 120 min blot shown in Figure 5 was stripped and re‐probed for FAT/CD36. MCT1 loading control (120 min) is the same as in Figure 5. Data were analyzed using an ordinary one‐way ANOVA at a given timepoint. Statistical significance was confirmed using a Tukey's post hoc test for a given timepoint. Statistical significance was accepted at *p* < 0.05. Data sharing a letter are not statistically different.

We sought to determine if FAT/CD36 concentration at the sarcolemma increased after UnAG injection as FAT/CD36 is a critical FA transporter in skeletal muscle. FAT/CD36 translocation to the sarcolemma was determined at 30 min post‐injection of insulin and at 120 min as these timepoints have previously shown a relative increase and return to baseline of sarcolemma concentrations of FAT/CD36, respectively (Snook et al., 2016). Thirty minutes post‐insulin injection the concentration of FAT/CD36 at the sarcolemma (Figure 6) was significantly increased relative to saline‐injected controls (*p* = 0.0442). At 120 min post‐injection, FAT/CD36 concentrations did not differ from saline‐injected controls (*p* = 0.8743). At 30 min post‐UnAG injection, sarcolemmal FAT/CD36 concentration did not differ significantly from saline‐injected controls (Figure 6; *p* = 0.1202), although it also did not differ from the insulin‐treated group. UnAG did not increase sarcolemmal FAT/CD36 concentration 120 min post‐injection relative to saline‐injected controls (*p* = 0.9917).

#### 3.4.2 FABPpm

**FIGURE 7:**
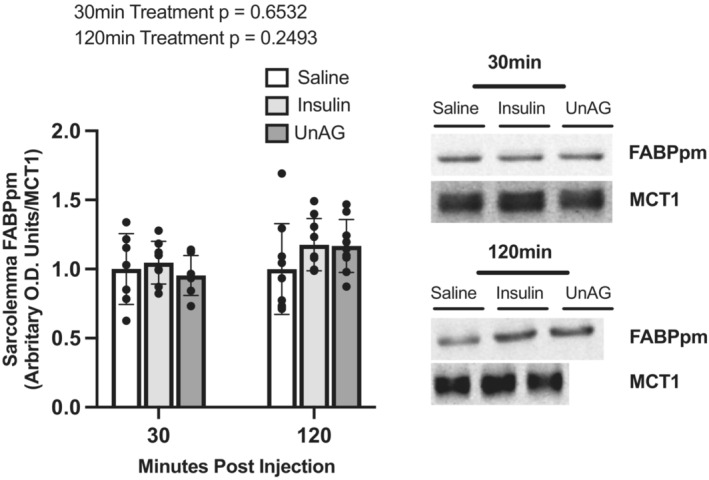
Isolated sarcolemma FABPpm concentrations in response to saline, insulin, and UnAG injections. Data are expressed as mean ± SD (*n* = 7–9). 30 min blot shown in Figure 6 was stripped and re‐probed for FABPpm. MCT1 loading control (30 min) is the same as in Figure 6. Data were analyzed using an ordinary one‐way ANOVA at each timepoint. Statistical significance was confirmed using a Tukey's post hoc test at a given timepoint. Statistical significance was accepted at *p* < 0.05. No statistical differences were found.

FABPpm concentrations were also determined at 30‐ and 120‐min post‐injection (Figure 7). Thirty minutes post‐insulin injection the concentration of FABPpm at the sarcolemma was not different relative to saline‐injected controls (*p* = 0.8542). At 120 min post‐insulin injection, FABPpm concentrations did not statistically differ from saline‐injected controls (*p* = 0.2392). Thirty minutes post‐UnAG injection FABPpm concentrations was not different relative to saline‐injected controls at the sarcolemma (*p* = 0.8628). UnAG did not statistically increase FABPpm concentrations at the sarcolemma 120 min post‐injection relative to saline‐injected controls (*p* = 0.2713).

### 3.5 In vivo, a single injection of unacylated ghrelin does not acutely affect whole‐body substrate utilization

**FIGURE 8:**
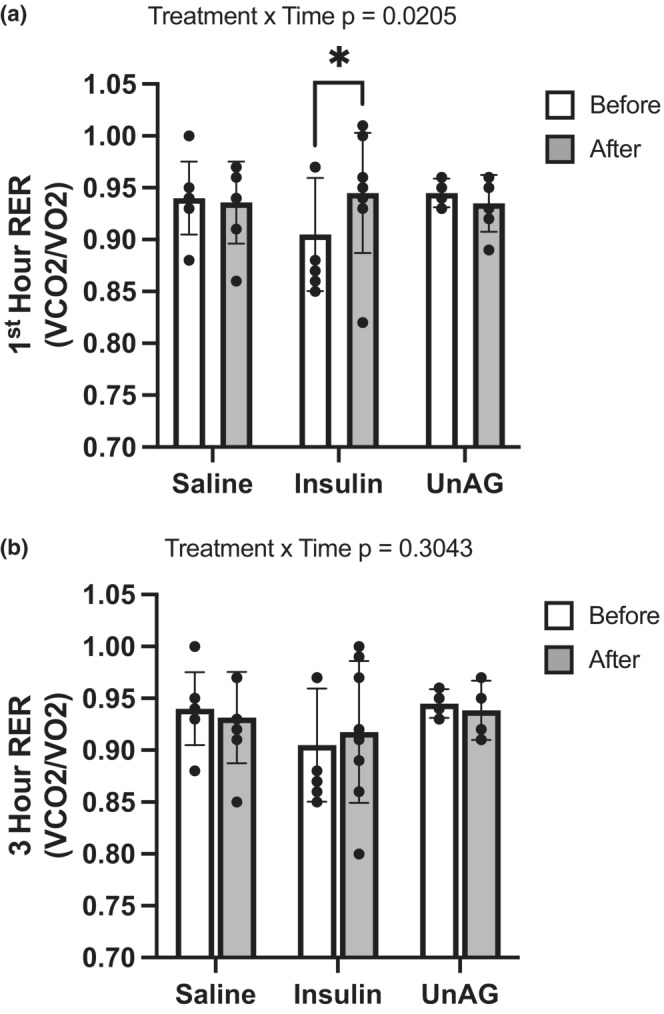
Respiratory exchange ratio (RER) before and after (1 h and 3 h) intraperitoneal injection of saline, insulin, or unacylated ghrelin. Data are expressed as mean ± standard deviation (*n* = 7–8). Insulin increased RER in the immediate 1 h post‐injection (a), but not over the entire 3 h (b). UnAG did not alter RER during the 3 h post‐injection. Data were analyzed using a two‐way ANOVA; when statistical significance was found a Bonferroni test was applied. Statistical significance was accepted at *p* < 0.05. Data denoted by an asterisk are statistically significant (*p* < 0.05).

We determined whether UnAG could influence whole‐body substrate utilization in vivo. Before injection, the mean RER values were determined from readings taken during the 3 h of the dark phase immediately prior to injection treatments for each animal. A 3 h period was also analyzed post‐injection to detect any effects that occurred immediately (i.e., within 1 h of treatment) and up to 3 h. Insulin increased RER (Figure 8) in the immediate 1 h post‐injection (*p* = 0.0205), but not cumulatively over the 3 h of analysis. Importantly, UnAG did not alter RER during either the immediate 1 h period post‐injection (Figure 8), or during the cumulative 3 h post‐injection.

## 4 DISCUSSION

In the present study, we sought to determine if (i) an initial 1 h exposure to UnAG would lead to a persistent increase in fatty acid oxidation 2–3 h later, (ii) if this increase in fatty acid oxidation was related to increased quantity of fat transporters (FAT/CD36 and FABPpm) at the sarcolemma, and (iii) if UnAGs' stimulation of fatty acid oxidation, as demonstrated ex vivo, could be detected at the whole‐body level using respirometry. The results of the current study demonstrate the novel finding of a persisting stimulus on fatty acid oxidation between 2 and 3 h after the initial UnAG exposure; however, this cannot be explained by persistent accumulation of fatty acid transporters on the sarcolemmal membrane.

### 4.1 UnAG and postprandial lipid metabolism

Skeletal muscle tissue is an important tissue in the clearance of fatty acids. It has been estimated that nearly all labeled palmitate from a test meal that reaches skeletal muscle is taken up, as determined by tracer entrapment in human forearm muscle (Bickerton et al., 2007; Evans et al., 2002). A known stimulus for the uptake of fatty acids is insulin, via the translocation of FAT/CD36 to the sarcolemma (Luiken et al., 2002; Snook et al., 2016; Stefanyk et al., 2012; van Oort et al., 2008). However, it is often overlooked that this stimulus subsides by 1 h (Snook et al., 2016; van Oort et al., 2008), and in fact, does not explain the main postprandial uptake and storage of fatty acids 2–3 h after consuming a meal (Dubois et al., 1998; Panzoldo et al., 2011; Snook et al., 2016). In the current study we have made the novel finding that a brief exposure to UnAG for 1 h can stimulate fatty acid oxidation for an additional 2 h after its removal. This provides evidence that UnAG has the potential to facilitate the utilization of meal‐derived fatty acids in skeletal muscle. In line with this finding, a single study in humans shows 30 min of UnAG infusion can lower circulating fatty acids after consumption of a mixed macronutrients test‐meal 2 h later, which is sustained for up to 6 h (Gauna et al., 2004). Although measurements of substrate utilization were not included in this study, these findings provide indirect evidence of a sustained effect of UnAG on fatty acid metabolism.

### 4.2 UnAG and regulation of skeletal muscle fatty acid oxidation

In the current study, we demonstrate that UnAG significantly increased muscle fatty acid oxidation ex vivo by ~50%. Importantly, this was still observed 2 h after removal of the initial ghrelin stimulus and was comparable to the other conditions of direct ghrelin exposure. Research on ghrelin, particularly UnAG, as a potential regulator of skeletal muscle fatty acid oxidation is relatively new. In humans, UnAG injection can reduce circulating fatty acid levels in a subsequent test‐meal and improve insulin sensitivity (Benso et al., 2012; Gauna et al., 2004). Our lab has previously demonstrated that UnAG is a significant stimulator of fatty acid oxidation in isolated rat skeletal muscle (Cervone et al., 2020; Kraft et al., 2019; Lovell et al., 2022). An important step in the regulation of muscle fatty acid oxidation is governed by CPT1 which regulates the entry of fatty acids into the mitochondria. Our lab has previously found that inhibition of CPT1 by etomoxir results in complete inhibition of UnAGs' stimulation of fatty acid oxidation, and its subsequent protective effects on glucose uptake under high‐saturated fat conditions (Cervone et al., 2020). This is strong evidence that UnAG exerts its protective effects on insulin sensitivity and glucose uptake at least in part by its ability to stimulation fatty acid oxidation. This is also consistent with findings in mice, in which the overexpression of UnAG preserves insulin signaling and reduces inflammation during 16 weeks of high‐fat feeding (Gortan Cappellari et al., 2016). Studies also show that increasing UnAG through injection or overexpression may exert various mitochondrial effects including increased expression of lipid metabolism enzymes in liver and muscle (Barazzoni et al., 2005), a reduction in high‐fat diet induced muscle reactive oxygen species and inflammation (Gortan Cappellari et al., 2016), improved mitochondrial function in adipose tissue from obese mice (Barazzoni et al., 2021), and the prevention of mitochondrial dysfunction during liver ischemia/reperfusion in a model of damaged rat liver (Rossetti et al., 2017). However, ghrelin kinetics are affected by macronutrient composition, as ghrelin is most strongly suppressed by protein and carbohydrate, and less so by fat (Foster‐Schubert et al., 2008; Monteleone et al., 2003; Overduin et al., 2005). Therefore, in the context of a meal consisting of mainly fat, our argument for requiring a persistent effect of UnAG on fatty acid oxidation for several hours may be less relevant. Thus, our findings of a persistent effect on fatty acid oxidation are more relevant to a typical mixed macronutrient meal.

While a growing body of research demonstrates that UnAG is a potential regulator of fatty acid metabolism in peripheral tissues, including skeletal muscle, the mechanisms regulating this response remain poorly understood. In the present study we aimed to address the possibility of UnAG‐mediated sarcolemmal trafficking of fatty acid transporters as a plausible explanation. However, UnAG did not persistently sustain signaling pathways regulating fatty acid translocation (e.g., AMPK, ACC) or increase the abundance of either FABPpm or FAT/CD36 on the sarcolemma at any point following UnAG exposure, challenging a key role for fatty acid transporters in UnAG‐mediated increases in fat oxidation. Additionally, while there was a trend for UnAG to rapidly induce FAT/CD36 sarcolemmal trafficking at 30 min (+36%; *p* = 0.1202), this is unlikely to contribute to the persistent effects observed in skeletal muscle palmitate oxidation that occurred 2 h later. Overall, this study is the first to demonstrate a persistent stimulus on fatty acid oxidation by UnAG. However, this finding cannot be explained by a persistent translocation of either FAT/CD36 or FABPpm to the sarcolemma.

### 4.3 Limitations and considerations

One general limitation is the supraphysiological dose of UnAG used in our interventions. Our dosage is in line with that previously used in our lab as well as others (Barazzoni et al., 2005; Cervone et al., 2020; Kraft et al., 2019; Lovell et al., 2022) and chosen to elicit a maximal response. We observed no differences in fatty acid oxidation between any of the isolated muscle UnAG exposure conditions despite different lengths of exposure and timing; thus, our supraphysiological dose may have elicited a maximal response and made it difficult to analyze any changes between treatment groups. Second, only male rats were utilized in these experiments given that our previous work was already established in males. Future studies should determine if our observed effects of UnAG on muscle FA metabolism are influenced by biological sex. Third, our finding of no effect on whole body substrate utilization (RER) with a single UnAG injection may question the physiological role of UnAG as a regulator of fatty acid oxidation. Hyperventilation is a stress response which can artificially increase the RER due to increased CO_2_ production. Thus, our protocol of handling the rats prior to injection, and the subsequent injection likely elicited a stress response, thereby inflating our RER values. This would make it difficult to identify any change in substrate utilization, especially considering our acute/brief window of analysis. We did observe a significant increase in RER in response to insulin injection, although this may have been influenced by several rats in this group starting off at a lower RER value. Fourth, GSVs were used to analyze the translocation of fat transporters to the sarcolemma. The sarcolemma represents ~50% of the total plasma membrane in skeletal muscle, but excludes the t‐tubule fraction. Therefore, we cannot rule out the possibility that fatty acid transporters may have accumulated in the t‐tubule fraction, which we would not have detected. Future studies could utilize a different fractionation technique to analyze fat transporter translocation to t‐tubules. Fifth, we cannot exclude the possibility that UnAG may have altered the intrinsic activity of the fatty acid transporters, thereby increasing fatty acid uptake. We also did not examine other putative fatty acid transporters, including FATP4, which has been shown to contribute to muscle fatty acid uptake and oxidation (Nickerson et al., 2009). Lastly, it is possible that the increase in fatty acid oxidation does not require the accumulation of fatty acid transporters at the cell surface, given that the increase in oxidation is relatively modest (~50%). This would suggest that the principle site of regulation lies downstream, potentially directly involving mitochondrial respiration or fatty acylcarnitine entry into the mitochondrion. However, this remains to be determined.

## 5 CONCLUSIONS

The hormonal signals that regulate postprandial fat metabolism in skeletal muscle are not well known. Insulin is known to cause the translocation of FAT/CD36 and FABPpm, but this is transient and cannot explain an increase in fatty acid oxidation 2–3 h after eating. The objective of this study was to determine if UnAG induces a persistent stimulation of fatty acid oxidation (i.e., for 2–3 h), and whether fatty acid transporter translocation to the sarcolemma is involved. Our results demonstrate that after removal of an acute UnAG stimulus, fatty acid oxidation remains stimulated for at least an additional 2 h. This sustained increase in oxidation is not associated with an increase in the sarcolemmal content of either FAT/CD36 or FABPpm. We conclude that UnAG may play an important role in the postprandial metabolism of fatty acids in skeletal muscle. Impaired UnAG signaling, as appears to occur in obesity, may potentially lead to the dysregulation of postprandial muscle fatty acid metabolism. At this point, there is no evidence for a role of fatty acid transporters. Further studies should examine the involvement of other transporters such as FATP4, changes in intrinsic transporter activity, as well as UnAG's effect on isolated mitochondrial respiration.

## FUNDING INFORMATION

This work was supported by the Natural Sciences and Engineering Research Council (NSERC) of Canada under Grant #400535 (DJD) and #400362 (GPH).

## CONFLICT OF INTEREST STATEMENT

No conflicts of interest declared.

## ETHICS STATEMENT

All animal procedures were reviewed and cleared by the University Animal Care Committee in accordance with the Canadian Council on Animal Care guidelines.
